# Supplementary material for: Genome-wide systematic identification of methyltransferase recognition and modification patterns
Source: Nat Commun. 2019 Aug 19;10:3311. doi: 10.1038/s41467-019-11179-9 (PMC6700114; doi:10.1038/s41467-019-11179-9)
Supplement: Supplementary file 1 — Supplementary information [file 41467_2019_11179_MOESM1_ESM.pdf]

Supplementary Information

Genome-wide systematic identification of methyltransferase recognition and modification patterns

Jensen et al.

Supplementary Figure 1

Graph showing the coverage (generated at std. parameters) as a function of the size of the insert (methyltransferase) in basepairs (bp).

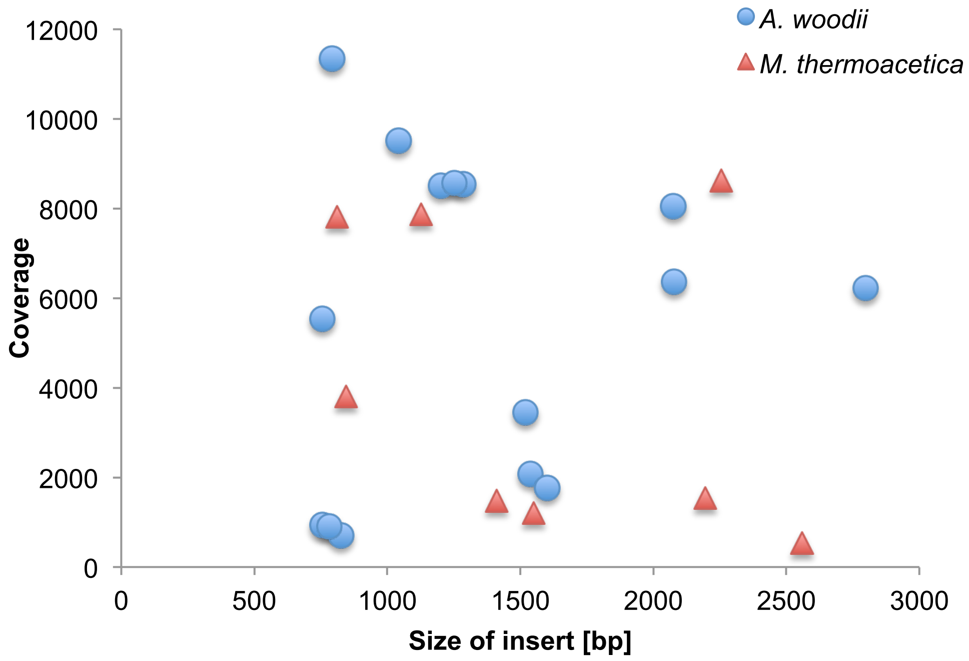

Supplementary Figure 2

Graph showing the coverage (generated at std. parameters) as a function of the DNA concentration (plasmid) in ng/µl coverage.

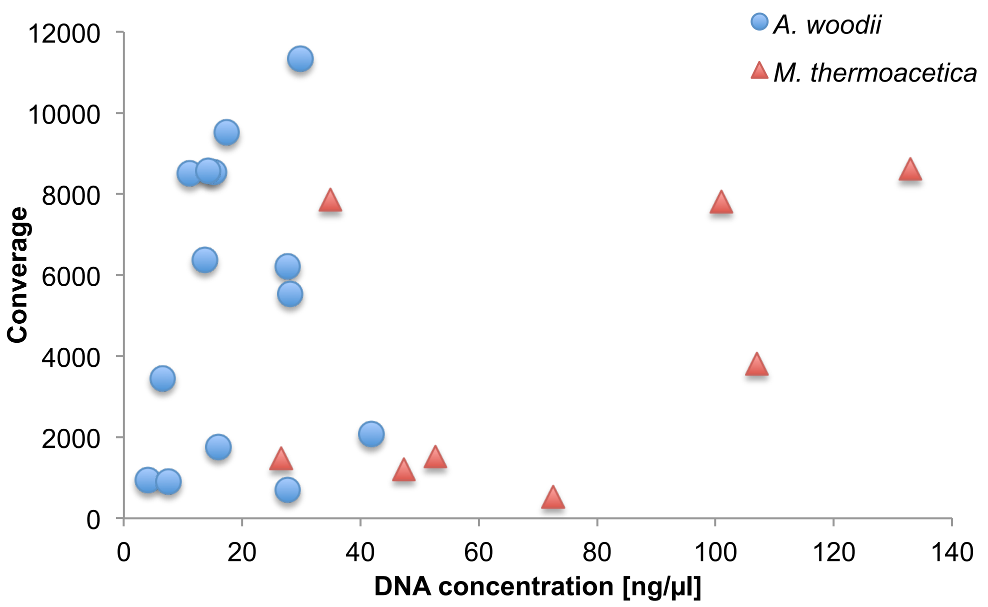

Supplementary Figure 3

Modification verification by restriction enzymes. Plasmid backbone is pRSFduetPA1lac*O*-1. Lanes marked with M are GeneRuler™ 1 kb DNA ladder (Thermo Scientific). Lanes 1 and 2 are MothHH_02467, lanes 5 and 6 are MothHH_01869, lanes 9 and 10 are MothHH_00029, and lanes 13 and 14 are Awo_c18590. Lanes 3,4,7,8,11,12,15 and 16 are the negative control *bgaB*.

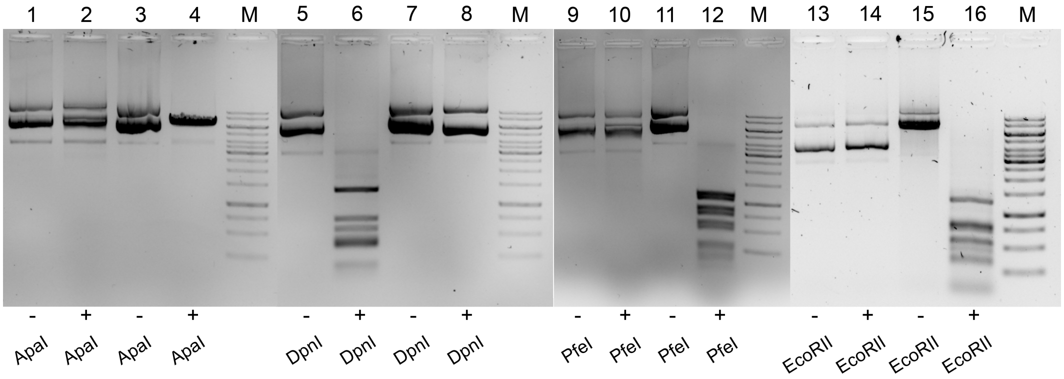

Supplementary Table 1

Numbers of restriction digestion sites on the plasmids tested by restriction digestion.

pRSFduetPA1lac*O*-1 with the following genes inserted	Size (bp)	ApaI GGGCCC	DpnI $G^{m6}ATC$	PfeI GAWTC	EcoRII CCWGG
MothHH_02467	6474	6			
MothHH_01869	5031		14		
MothHH_00029	5346			21	
Awo_c18590	5504				27
bgaB (negative cont.)	6302	1	14	12	14

Supplementary Figure 4

Sequence of the motif-cassette with motifs for assessing the methyltransferases of *M. thermoacetica*.

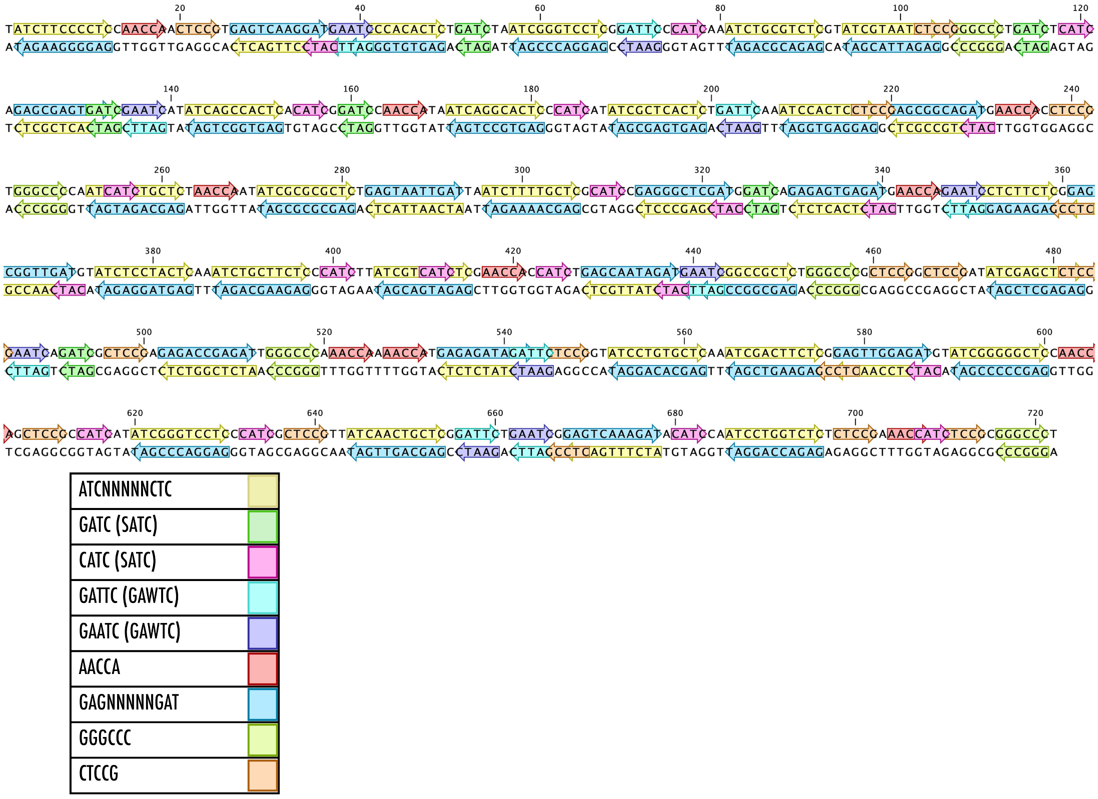

Raw sequence

TATCTTCCCCTCCAACCAACTCCGTGAGTCAAGGATGAATCCCACACTCTGATCTAAT
CGGGTCCTCGGATTCCCATCAAATCTGCGTCTCGTATCGTAATCTCCGGGCCCTGATC
TCATCAGAGCGAGTGATCGAATCATATCAGCCACTCACATCGGATCCAACCATAATC
AGGCACTCCCATCATATCGCTCACTCTGATTCAAATCCACTCCTCCGAGCGGCAGATG
AACCACCTCCGTGGGCCCCAATCATCTGCTCTAACCAATATCGCGCGCTCTGAGTAAT
TGATTAATCTTTTGCTCGCATCCGAGGGCTCGATGGATCAGAGAGTGAGATGAACCA
GAATCCTCTTCTCGGAGCGGTTGATGTATCTCCTACTCAAATCTGCTTCTCCCATCTT
ATCGTCATCTCGAACCACCATCTGAGCAATAGATGAATCGGCCGCTCTGGGCCCGCTC
CGGCTCCGATATCGAGCTCTCCGAATCAGATCGCTCCGAGAGACCGAGATTGGGCCCA
AACCAAAACCATGAGAGATAGATTCTCCGGTATCCTGTGCTCAAATCGACTTCTCGG
AGTTGGAGATGTATCGGGGGCTCCAACCAGCTCCGCCATCATATCGGGTCCTCCCATC
GCTCCGTTATCAACTGCTCGGATTCTGAATCGGAGTCAAAGATACATCCAATCCTGGT
CTCTCTCCGAAACCATCTCCGCGGGCCCT -3´

Supplementary Figure 5

Sequence of the motif cassette for assessing the methyltransferases of *A. woodii*.

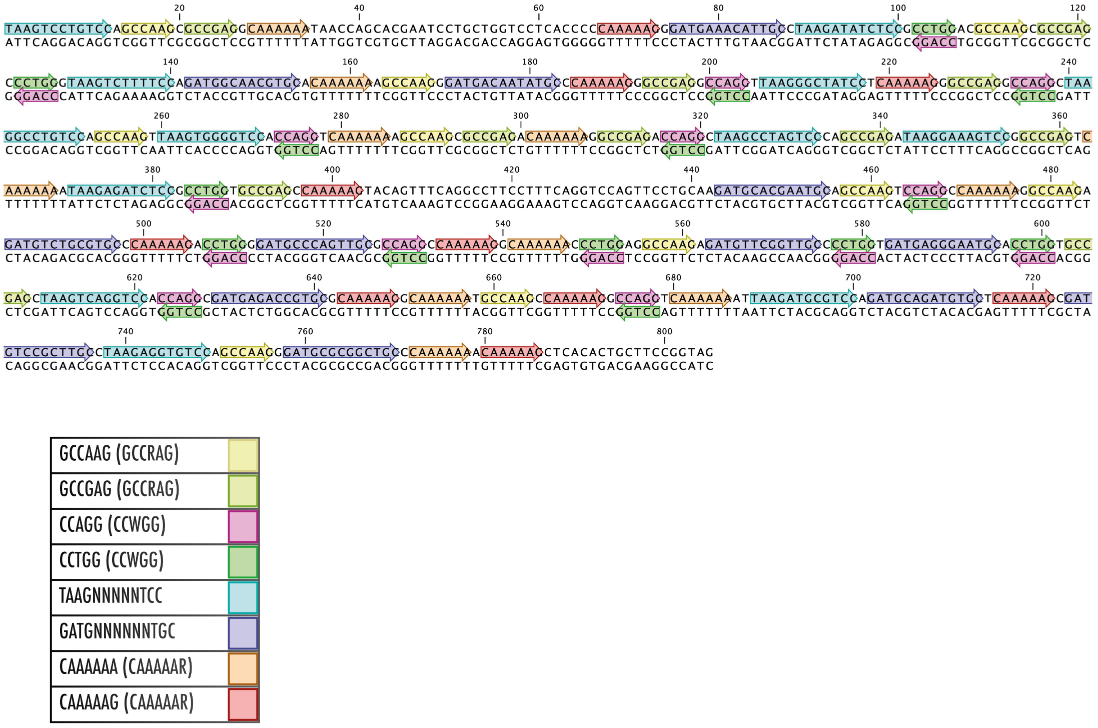

Raw sequence

TAAGTCCTGTCCAGCCAAGCGCCGAGGCAAAAAATAACCAGCACGAATCCTGCTGGT
CCTCACCCCCAAAAAGGGATGAAACATTGCCTAAGATATCTCCGCCTGGACGCCAAGC
GCCGAGCCCTGGGTAAGTCTTTTCCAGATGGCAACGTGCACAAAAAAAGCCAAGGGA
TGACAATATGCCCAAAAAGGGCCGAGGCCAGGTTAAGGGCTATCCTCAAAAAGGGCC
GAGGCCAGGCTAAGGCCTGTCCAGCCAAGTTAAGTGGGGTCCACCAGGTCAAAAAAA
GCCAAGCGCCGAGACAAAAAAGGCCGAGACCAGGCTAAGCCTAGTCCCAGCCGAGAT
AAGGAAAGTCCGGCCGAGTCAAAAAAATAAGAGATCTCCGCCTGGTGCCGAGCCAAA
AAGTACAGTTTCAGGCCTTCCTTTCAGGTCCAGTTCCTGCAAGATGCACGAATGCAGC
CAAGTCCAGGCCAAAAAAGGCCAAGAGATGTCTGCGTGCCCAAAAAGACCTGGGGAT
GCCCAGTTGCGCCAGGCCAAAAAGGCAAAAAACCCTGGAGGCCAAGAGATGTTCGGT
TGCCCCTGGTGATGAGGGAATGCACCTGGTGCCGAGCTAAGTCAGGTCCACCAGGCG
ATGAGACCGTGCGCAAAAAGGCAAAAAATGCCAAGCCAAAAAGGCCAGGTCAAAAAA
ATTAAGATGCGTCCAGATGCAGATGTGCTCAAAAAGCGATGTCCGCTTGCCTAAGAG
GTGTCCAGCCAAGGGATGCGCGGCTGCCCAAAAAAACAAAAAGCTCACACTGCTTCC
GGTAG -3´

Supplementary Table 2

Media composition for cultivating *M. thermoacetica*, according Daniel et al. [62].

Components	Concentration
$AlK(SO_4)_2{\cdot}12H_2O$	50 µg/l
Biotin	0.1 mg/l
$CaCl_2{\cdot}2H_2O$	50 mg/l
Calcium D-(+)-panthothenate	0.25 mg/l
$Co(NO_3)_2{\cdot}$ $6H_2O$	0.5 mg/l
$CuSO_4{\cdot}5H_2O$	0.05 mg/l
Cystein-HCl	158 mg/l
$FeSO_4{\cdot}7H_2O$	0.5 mg/l
Folic acid*	0.1 mg/l
Fructose	5 g/l
H_3BO_3	0.05 mg/l
KH_2PO_4	500 mg/l
Lipoic acid	0.15 mg/l
$MgCl_2$	330 mg/l
$MnSO_4{\cdot}H_2O$	2.5mg/l
$Na_2MoO_4{\cdot}2H_2O$	0.05 mg/l
NaCl	400 mg
$NaHCO_3$	3.5 g/l
NH_4Cl	400 mg/l
$NiCl_2{\cdot}6H_2O$	0.25 mg/l
Nicotinic acid	0.25 mg/l
Nitrilotriacetate	7.5 mg/l
p-Aminobenzoic acid	0.25 mg
Pyridoxine hydrochloride	0.05 mg/l
Resazurin	1 mg/l
Riboflavin	0.25 mg/l
Thiamine HCL	0.25 mg/l
Vitamin B12 (Cyanocobalamin)	0.25 mg/l
Yeast extract	1 g/l
$ZnCl_2$	0.5 mg/l
