# Supplementary material for: Genome-wide systematic identification of methyltransferase recognition and modification patterns
Source: Nat Commun. 2019 Aug 19;10:3311. doi: 10.1038/s41467-019-11179-9 (PMC6700114; doi:10.1038/s41467-019-11179-9)
Supplement: Supplementary file 3 — Description of Additional Supplementary Files [file 41467_2019_11179_MOESM3_ESM.pdf]

Description of Additional Supplementary Files

File Name: Supplementary Data 1
Description: Raw data from methylation detection. Assembly of data presented in motif_summary.csv files

File Name: Supplementary Data 2
Description: List of strains and plasmids used in this study

File Name: Supplementary Data 3
Description: List of primers used in this study
